# Fermented Foods as Functional Systems: Microbial Communities and Metabolites Influencing Gut Health and Systemic Outcomes

**DOI:** 10.3390/foods14132292

**Published:** 2025-06-28

**Authors:** Inmyoung Park, Mohamed Mannaa

**Affiliations:** 1 School of Food and Culinary Arts, Youngsan University, Busan 48015, Republic of Korea; 2 Department of Plant Pathology, Faculty of Agriculture, Cairo University, Giza 12613, Egypt; 3 Department of Integrated Biological Science, Pusan National University, Busan 46241, Republic of Korea

**Keywords:** functional nutrition, fermentation microbiota, gut microbiome, microbiome modulation, diet–microbiome interactions

## Abstract

Fermented foods represent an intricate ecosystem that delivers live microbes and numerous metabolites, influencing gut health. In this review, we explore how complex microbial communities and metabolites generated during food fermentation modulate the gut microbiome and affect human health. We discuss fermentation-induced biochemical transformations, including enhanced fiber fermentability; nutrient availability; and the synthesis of bioactive metabolites such as short-chain fatty acids, exopolysaccharides, bacteriocins, and modified polyphenols. We describe the dynamic microbial ecology of fermented foods, influenced by ingredient variations, highlighting its effect on health-related metabolic outcomes. Fermented products when consumed transiently introduce beneficial microbes and bioactive compounds into the gut, thereby boosting microbial diversity, resilience, and barrier function. We review clinical and preclinical studies to substantiate the roles of fermented foods in immune regulation, metabolic homeostasis, cognitive function, and inflammation mitigation. Individual variability in response to fermented foods has been emphasized, underscoring the potential for personalized nutrition strategies informed by advanced omics technologies. By integrating microbial ecology, metabolomics, and clinical evidence, this review positions fermented food intake as a strategic dietary intervention for microbiome modulation and health promotion.

## 1. Introduction

Fermented foods have been an essential component of human diets since ancient times; although the fermentation process was initially developed for food preservation, it is valued for enhancing flavor, texture, and nutritional benefits [1]. Fermentation products such as yogurt, kefir, kimchi, sauerkraut, tempeh, and miso are an integral part of culinary and medicinal practices across diverse cultures. Beyond preservation, fermentation substantially transforms food matrices, enhancing digestibility, improving nutrient bioavailability, and introducing beneficial microbial communities, diverse enzymes, and various bioactive metabolites [2].

In recent decades, fermented foods have garnered renewed scientific interest, largely driven by the growing evidence of their beneficial effects on the human gut microbiome [3]. The gut microbiome, comprising trillions of diverse microorganisms, including bacteria, archaea, fungi, and viruses, exerts profound effects on human physiology, metabolism, immune regulation, and overall health [4,5]. Dietary approaches aimed at positively modulating the gut microbiome are being recognized as pivotal for health promotion and disease prevention.

In contrast to isolated probiotic supplements, fermented foods contain complex microbial ecosystems that deliver diverse live microorganisms and bioactive metabolites, which uniquely interact with and influence the resident gut microbiota. Clinical evidence suggests that regular consumption of fermented foods can substantially enhance gut microbial diversity, improve intestinal barrier function, and modulate systemic inflammation, thereby positively influencing various chronic health conditions [6,7]. However, there is considerable variability in individual responses to fermented foods, regulated by factors such as genetics, baseline microbiota composition, dietary habits, and environmental exposure. This variability underscores the need for personalized nutritional strategies based on microbiome profiling [8].

**Figure 1:**
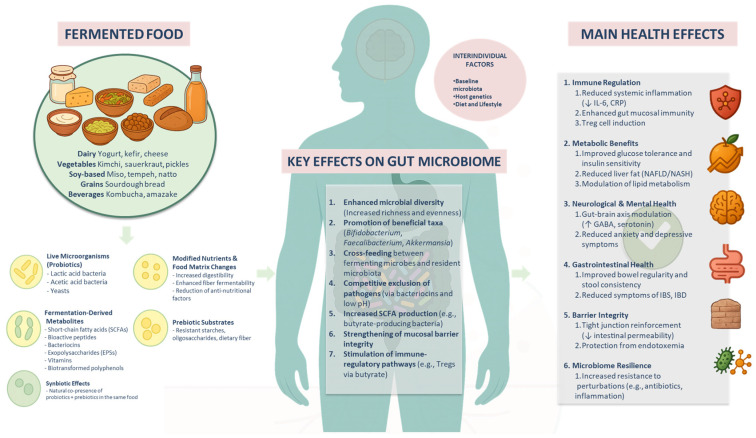
Overview of the key mechanisms through which fermented foods influence gut microbiota and host health. Fermented foods—including dairy (e.g., yogurt and kefir), vegetables (e.g., kimchi and sauerkraut), soy-based products (e.g., miso and tempeh), grain-based products (e.g., sourdough bread), and beverages (e.g., kombucha and amazake)—deliver live beneficial microbes, fermentation-derived metabolites (e.g., organic acids such as acetic acid, exopolysaccharides, bacteriocins, and vitamins), modified nutrients, and prebiotic substrates. These components interact with the host gut microbiome by enhancing microbial diversity; promoting the growth of beneficial taxa (e.g., *Bifidobacterium*, *Faecalibacterium*, and *Akkermansia*); facilitating cross-feeding; suppressing the growth of pathogens; and promoting gut microbiota-derived short-chain fatty acid (SCFA) production, mucosal integrity, and immune modulation. These microbiome-mediated effects contribute to systemic health outcomes including immune regulation, metabolic improvement, neurological and gastrointestinal health, barrier function, and microbiome resilience. Interindividual factors such as baseline microbiota, host genetics, and lifestyle influence response to fermented food interventions.

In this review, we critically evaluate fermented foods as dynamic systems that shape the human gut microbiome and enhance health outcomes. We first discuss the biochemical transformations that occur during fermentation—including microbial enzyme activity—and their influence on food nutritional quality, such as enhanced fiber accessibility, bioactive compound production, and improved digestibility. Subsequently, we focus on fermented food microbiomes, describing their microbial diversity and ecological dynamics, and examine how these microbes and metabolites interact with the gut microbiota upon consumption, thereby affecting microbial composition, resilience, and metabolic health. Furthermore, we consolidate evidence from current clinical and preclinical studies linking fermented food consumption to improvements in immune function, metabolic regulation, inflammation control, and cognitive health. Recognizing interindividual differences, we discuss the prospects of personalized nutritional approaches based on microbiome profiling and precision fermentation technologies. We highlight key knowledge gaps and propose future research directions to optimize fermented foods as a microbiome-targeted nutritional intervention. Finally, we present an integrated conceptual overview to summarize the multidimensional interactions among fermented foods, the gut microbiome, and systemic health (Figure 1). Although this review does not differentiate between naturally fermented and artificially inoculated foods, it is important to recognize that differences in fermentation methods may influence microbial diversity and metabolite composition. Future studies could compare their distinct impacts on health-related outcomes.

## 2. Fermented Food Characteristics: Beyond Live Microbes

Traditionally, fermented foods have been recognized primarily for their probiotic properties, which are largely attributed to the content of live beneficial microorganisms such as lactic acid bacteria (LAB), bifidobacteria, and yeasts [9]. However, recent research indicates that the health-promoting characteristics of fermented foods are not limited to the live microbial communities. During fermentation, complex biochemical transformations occur within food matrices, profoundly modifying their nutritional composition, enhancing nutrient bioavailability, and producing a diverse array of bioactive metabolites, which collectively play a substantial role in modulating the gut microbiome composition and influencing host health outcomes [10,11].

### 2.1. Enhanced Dietary Fiber Bioavailability and Nutrient Accessibility

Dietary fibers, particularly complex polysaccharides, are pivotal substrates for the gut microbiota, selectively supporting beneficial bacterial growth. However, the structural complexity of these fibers often limits their fermentability and accessibility to gut microbes. Fermentation processes address this limitation by enzymatically breaking down complex carbohydrates into simpler sugars, oligosaccharides, and more accessible polysaccharides, thereby enhancing the fermentability and prebiotic potential of the fibers [12,13]. For example, during the fermentation of cabbage into sauerkraut or kimchi, the levels of structural polysaccharides and anti-nutritional factors, such as glucosinolates and phytates, are substantially reduced. This biochemical transformation results in an increase in soluble fiber content and improved digestibility, which in turn supports the proliferation and metabolic activity of beneficial gut microbes upon consumption [14,15]. Similarly, in cereal fermentations, such as in sourdough bread production, LAB and yeasts substantially reduce the level of phytic acid, a strong chelator of minerals, thereby enhancing mineral bioavailability and improving overall nutritional quality [16].

### 2.2. Generation of Bioactive Metabolites During Fermentation

Fermentation processes produce various organic acids such as lactic, acetic, gluconic, and glucuronic acids, particularly in fermented dairy and kombucha beverages. Additionally, fermentation generates bioactive compounds including bacteriocins (e.g., nisin and plantaricin), bioactive peptides—such as antihypertensive peptides (Val-Pro-Pro (VPP) and Ile-Pro-Pro (IPP)) and antimicrobial peptides (lactoferricin and casocidin-I)—extracellular polysaccharides (EPSs) with notable prebiotic and immunomodulatory properties, and modified polyphenolic compounds like gallic acid derivatives. These metabolites significantly enhance the nutritional and health-promoting properties of fermented foods [10,11].

Short-chain fatty acids (SCFAs), predominantly acetate, propionate, and butyrate, are primarily produced in the gut by resident microbiota fermenting dietary fibers. SCFAs serve as energy sources to colonocytes, fortify gut barrier integrity, modulate inflammatory responses, and regulate metabolism and appetite [17,18]. Although fermented foods generally contain modest levels of SCFAs, regular consumption of fermented foods such as kefir [19] and kimchi [20] provides dietary substrates and beneficial microbes that subsequently stimulate the gut microbiota to produce larger quantities of SCFAs, thereby enhancing gut and systemic health.

Bacteriocins are antimicrobial peptides primarily synthesized by LAB. They exert potent antimicrobial effects against pathogens and spoilage microorganisms, helping maintain microbiota homeostasis and enhancing food safety and shelf life. Fermented foods depicted in Figure 1, including yogurt, kefir, cheese, kimchi, and sauerkraut, frequently harbor bacteriocin-producing LAB strains, making these foods valuable for gastrointestinal health and protection against enteric pathogens [21,22].

Exopolysaccharides (EPSs), high-molecular-weight polysaccharides secreted by certain fermentation microbes, have considerable prebiotic and immunomodulatory properties. EPSs produced by LAB in fermented dairy products such as kefir and yogurt enhance mucosal barrier integrity, modulate intestinal immunity, selectively support beneficial gut microbiota, and exert cholesterol-lowering effects [23,24].

Fermentation also leads to the microbial biotransformation of plant-derived polyphenols, significantly enhancing their bioavailability and bioactivity. In products such as kombucha, tempeh, and miso, microbial enzymes transform complex polyphenolic structures into smaller and more bioavailable metabolites with improved antioxidant, antimicrobial, and anti-inflammatory effects. These modified polyphenolic metabolites beneficially interact with the gut microbiota, promoting microbial diversity and contributing to metabolic health [25].

### 2.3. Prebiotic and Synbiotic Properties of Fermented Foods

Fermented foods also function naturally as prebiotic and synbiotic systems. By virtue of their biochemical transformations, fermented foods often contain high concentrations of fermentable substrates such as oligosaccharides and modified polysaccharides, selectively stimulating the growth and activity of beneficial gut microbes such as *Bifidobacterium* and *Lactobacillus* species [26]. For instance, fermented soybean products such as tempeh and natto contain increased levels of bioactive oligosaccharides that specifically foster beneficial gut microbial communities, thus enhancing microbial diversity and promoting gut metabolic health [27]. Additionally, the simultaneous presence of probiotics (live beneficial microorganisms) and prebiotic substances (substrates that probiotics selectively utilize) in fermented foods exemplifies their natural synbiotic properties, exerting a synergistic effect that enhances microbiome modulation beyond the individual capabilities of probiotics or prebiotics alone [28].

### 2.4. Vitamin and Bioactive Peptide Production

Fermentation also markedly increases the levels of certain vitamins (e.g., the vitamin B complex and vitamin K) and bioactive peptides within food matrices. The bioactive peptides generated through microbial proteolytic activities exhibit antihypertensive, antioxidant, immunomodulatory, and antimicrobial properties. Fermented dairy products such as kefir and yogurt are particularly rich in bioactive peptides derived from milk proteins, contributing to their health-promoting potential [29].

Vitamin synthesis is another valuable aspect of fermentation, as certain LAB and yeasts can synthesize essential vitamins, thereby enhancing the nutritional value of the final product. For example, fermentation processes in products such as kefir, yogurt, and fermented vegetables significantly increase the bioavailability of vitamins such as B12, folate, and vitamin K2, which are essential for metabolic, neurological, and cardiovascular health [30].

A wide range of fermented foods contain diverse microbial strains and bioactive metabolites that possess multiple health benefits. Table 1 summarizes the representative fermented foods, their dominant microbial constituents, key fermentation-derived metabolites, and health-related effects based on clinical and preclinical study data.

## 3. Fermented Food Microbiomes and Their Dynamics

Fermented foods harbor complex microbial ecosystems comprising diverse populations of beneficial microbes such as LAB, acetic acid bacteria, yeasts, and molds. These microbial communities are not static; rather, they continuously evolve during the fermentation process and respond dynamically to intrinsic factors (e.g., substrate composition, pH changes, and nutrient availability) and extrinsic factors (e.g., temperature, oxygen levels, and fermentation duration) [45,46]. An in-depth understanding of fermented food microbiomes and their ecological dynamics is essential for optimizing their quality, consistency, and safety as well as for accurately predicting and maximizing their health-promoting effects upon consumption.

### 3.1. Diversity and Composition of Fermented Food Microbiomes

Advancements in molecular techniques, particularly next-generation sequencing and metagenomics, have considerably improved our understanding of the microbial diversity of fermented foods. Different fermentation types are characterized by distinct microbial consortia influenced primarily by the initial substrates, environmental conditions, and traditional or introduced starter cultures.

Traditional fermented vegetables, such as Korean kimchi and European sauerkraut, are primarily dominated by LAB, including species within the genera *Lactobacillus*, *Leuconostoc*, *Weissella*, and *Pediococcus* [14,47]. In kimchi fermentation, an initial dominance of *Leuconostoc mesenteroides* is typically observed, establishing an environment conducive to the subsequent growth of acid-tolerant species such as *Lactobacillus plantarum* and *Lactobacillus brevis* [48]. This structured microbial succession is critical for developing the distinct sensory and nutritional characteristics of kimchi and for enriching it with vitamins, lactic acid, and various bioactive peptides [20].

Recent studies have highlighted that subtle changes in ingredient formulations markedly influence microbial community structures and metabolite profiles. For example, adding fresh seafood, particularly gizzard shad, to kimchi fermentation can selectively promote the growth of beneficial microbes such as *Leuconostoc rapi*, a bacterium associated with improved flavor and antioxidant production and reduced undesirable acidity caused by *Lactobacillus sakei* during later fermentation stages [49].

Similarly, in Korean fermented soybean products, such as doenjang (fermented soybean paste) and gangjang (soy sauce), ingredient modifications considerably reshape the microbiome. The addition of coriander during gangjang fermentation reduces the presence of halophilic *Chromohalobacter beijerinckii*, a bacterium known to produce biogenic amines. This reduction corresponds to a substantial decrease in the levels of biogenic amines, notably histamine and tyramine, which enhances product safety and quality [50].

In doenjang, supplementation of herbs such as peppermint and Korean mint promotes beneficial microbial shifts by suppressing the growth of potentially harmful bacteria, such as *Sphingomonas* and *Pantoea*, while enriching beneficial microbes, such as *Saccharopolyspora* and *Buttiauxella*, which are known for their beneficial enzymatic and antimicrobial activities [51]. Such strategic changes in ingredients underscore the potential of targeted microbial modulation for optimizing both product safety and health-promoting functionalities.

Fermented dairy products, including yogurt, kefir, and artisanal cheeses, harbor complex and diverse microbial consortia. Yogurt represents a synergistic partnership of *Streptococcus thermophilus* and *Lactobacillus delbrueckii* subsp. *bulgaricus*. In contrast, kefir is characterized by greater microbial complexity involving multiple bacterial species (*Lactobacillus kefiranofaciens*, *Lactococcus lactis*, and *Leuconostoc mesenteroides*) co-existing symbiotically with yeast species such as *Saccharomyces cerevisiae* and *Kluyveromyces marxianus* [36,52]. These microbial communities markedly influence the product characteristics, including texture, flavor, and probiotic potential.

Kombucha, a popular fermented tea beverage, represents another fascinating example of complex microbial interactions. Its characteristic microbial community comprises acetic acid bacteria, primarily *Komagataeibacter xylinus* (previously classified within the genus *Gluconacetobacter*), and diverse yeasts, such as *Zygosaccharomyces*, collectively forming a cellulose-based biofilm known as a symbiotic culture of bacteria and yeast. The unique metabolic interactions within kombucha microbiomes produce distinct bioactive metabolites such as acetic, gluconic, and glucuronic acids, contributing to the sensory attributes and potential health benefits of kombucha [53].

### 3.2. Ecological and Metabolic Dynamics During Fermentation

The fermentation process involves distinct and dynamic ecological successions driven by microbial competition and cooperation, substrate availability, oxygen depletion, and progressive acidification. Initially, a diverse range of microorganisms colonize the fermentation substrate; however, as conditions become more acidic and anaerobic, selective pressures lead to dominance by specific microbial groups adapted to these conditions [54].

During vegetable fermentation, the aerobic microbes present in the initial stages consume residual oxygen, facilitating the subsequent establishment of anaerobic LAB. LAB-driven lactic acid production lowers pH, suppresses the growth of pathogenic organisms, and promotes the selective growth of desirable acid-tolerant microbes [55]. Such ecological shifts directly influence the sensory characteristics, shelf life, and nutritional value of the final product, emphasizing the importance of controlled ecological dynamics to achieve consistent fermented food quality.

## 4. Effect of Fermented Food Microbiomes on Human Gut Microbiota

The consumption of fermented foods transiently introduces complex microbial communities into the gastrointestinal tract, dynamically interacting with the resident gut microbiota. Although microbes from fermented foods generally do not exhibit long-term colonization, their temporary presence substantially influences microbial diversity, metabolic activity, and ecological resilience within the gut ecosystem [56,57].

Regular intake of fermented products, including yogurt, kefir, kimchi, and sauerkraut, has consistently been associated with beneficial alterations in the gut microbiota. Notably, increased microbial diversity and enrichment of health-promoting taxa such as *Bifidobacterium*, *Lactobacillus*, and *Akkermansia* species have been frequently reported [58,59,60]. These beneficial microbes enhance the gut barrier function, modulate immune responses, and support anti-inflammatory processes.

Recent meta-omics and clinical studies have significantly advanced our understanding of how fermented food consumption modulates the gut microbiota composition. Amplicon sequencing and shotgun metagenomic analyses have identified beneficial taxa such as *Komagataeibacter* (formerly *Gluconacetobacter*) and *Zygosaccharomyces* species as dominant microbes in kombucha fermentations, highlighting their potential functional roles and probable contributions to gut health upon consumption [61]. Complementary evidence from animal studies has demonstrated beneficial microbial shifts following kombucha administration. For instance, Jung et al. [62] reported a significant reduction in the abundance of pro-inflammatory taxa including *Erysipelotrichia*, *Turicibacter*, and *Clostridium*, accompanied by an increase in the abundance of beneficial genera such as *Lactobacillus* and *Mucispirillum* in mice with nonalcoholic fatty liver disease (NAFLD). These microbial changes correlated directly with improved liver health and decreased liver fat accumulation, underscoring the role of kombucha in metabolic homeostasis via gut microbiome modulation [62]. More recently, a controlled clinical study confirmed that short-term kombucha consumption in humans enriched the gut microbiota with SCFA-producing taxa, notably *Weizmannia coagulans*, a probiotic strain typically associated with kombucha fermentation. Although the observed microbiome shifts were modest owing to a short intervention duration and high interindividual variability, this study highlights the potential of kombucha to enhance microbial metabolism and support a healthy gut microbiome through increased SCFA production [63].

Fermented foods also contribute to gut microbial stability and resilience by suppressing the growth of pathogenic microorganisms. Specifically, bacteriocins produced by LAB, such as nisin by *Lactococcus lactis* and plantaricin by *Lactobacillus plantarum*, exert selective growth-inhibitory effects against enteric pathogens, thereby protecting gut microbiome integrity [64]. Furthermore, bioactive metabolites resulting from fermentation, particularly modified dietary fibers and polyphenols (described comprehensively in Section 2), improve substrate availability and bioactivity, further supporting the proliferation of beneficial gut microbiota [44,65].

Advances in the understanding of fermented food microbiomes are fueling innovations in precision fermentation, enabling targeted modulation of microbiota and their metabolites for personalized nutritional interventions. By selecting specific microbial consortia and fermentation conditions, precise fermentation approaches have the potential to optimize fermented products tailored to individual microbiome compositions and health objectives [66,67].

In summary, fermented foods considerably influence the gut microbiota through transient interactions among microbes, promotion of ecological resilience, suppression of pathogen growth, and enhancement of microbial diversity. These multidimensional interactions make fermented foods powerful dietary components for personalized microbiome management, extending their health-promoting effects beyond those of conventional probiotic supplements. Leveraging insights from meta-omics and targeted fermentation strategies can further enhance these benefits and foster tailored microbiome-targeted nutritional approaches.

## 5. Interindividual Variability in Response to and Precision Nutrition Opportunities with Fermented Foods

Fermented food intake represents a promising approach for modulating the gut microbiota and enhancing systemic health, delivering not only live microbes but also diverse metabolites within a complex food matrix. While these benefits are being increasingly recognized, individual responses to fermented food consumption vary substantially owing to the complex interactions among host genetics, baseline microbiota composition, dietary patterns, and lifestyle factors. Such variability influences microbial engraftment, metabolite utilization, and subsequent physiological outcomes.

Recent studies have underscored the individualized effects of fermented foods. Zmora et al. [68] demonstrated that probiotic colonization in the human gut is strongly individualized and is influenced by pre-existing microbiome structures and host characteristics, resulting in distinct colonization patterns. Similarly, Korem et al. [69] highlighted how unique microbiome profiles predict differential glycemic responses among individuals consuming identical foods, further emphasizing the need for tailored nutritional approaches. Zeevi et al. [70] expanded upon this by developing predictive machine learning models that integrate microbiome, dietary, and clinical data to accurately predict individualized metabolic responses, thereby supporting the development of personalized dietary strategies.

The concept of precise nutrition has been increasingly facilitated through advancements in integrative omics technologies including metagenomics, metabolomics, and transcriptomics. Such approaches allow for the detailed characterization of host–microbe interactions, providing a deeper understanding of how fermented foods influence specific biological pathways relevant to health. Machine learning and high-resolution multi-omics datasets are being used to tailor fermented food interventions to individual microbiota compositions, focusing on key health-related pathways such as SCFA synthesis, bile acid metabolism, immune modulation, and metabolic health [71].

Recent advances have facilitated the integration of machine learning and multi-omics technologies to decipher complex microbial dynamics and metabolic pathways in fermented food systems. For instance, the Omics Database of Fermentative Microbes is a curated genomic and metagenomic resource that encompasses microorganisms commonly associated with traditional fermented foods, including LAB, yeasts, and molds. This database enables precise taxonomic assignment, genomic comparisons, and the evaluation of potential starter strains based on omics data, thus supporting targeted strain selection and improved fermentation control [72]. Complementing this, Li et al. [73] applied machine learning algorithms, including logistic regression, random forest, and K-nearest neighbors, along with metagenomic and flavoromic data, to classify abnormal versus optimal fermentation outcomes in sauce-flavor Baijiu. In their study, SHapley Additive exPlanations values helped unveil key microbial and metabolic markers distinguishing fermentation states, while ecological modeling provided insights into community assembly mechanisms under different environmental conditions. These integrative approaches serve as powerful tools for predicting fermentation performance, diagnosing microbial imbalances, and tailoring fermented food production to improve quality and reproducibility.

Innovative precision fermentation techniques enable targeted microbiome modulation through tailored microbial consortia or optimized fermentation conditions, allowing for the design of fermented products that directly address specific health conditions, including metabolic syndrome, inflammatory bowel diseases, and neurological disorders [74,75]. Qian and Ho [76] emphasize that beyond identifying beneficial microbial taxa, understanding the ecological interactions among microbes and their metabolites is critical for developing next-generation precision nutritional interventions.

Future research must include robust clinical trials specifically designed to assess the microbial viability, safety profiles, and dose–response relationships of fermented food interventions while accounting for interindividual variability. Longitudinal studies integrating multi-omics analyses to monitor microbiota dynamics, metabolite shifts, and clinical biomarkers are essential for validating personalized approaches. Additionally, the development of comprehensive databases linking microbial taxa and metabolites to specific health outcomes is critical for advancing regulatory standards and ensuring effective therapeutic applications [77,78].

By addressing interindividual variability through advanced omics and precision fermentation technologies, fermented foods can be strategically used as personalized dietary interventions to maximize their potential in microbiome-driven health promotion.

## 6. Clinical and Preclinical Evidence Supporting the Health Benefits of Fermented Foods

Substantial preclinical and human study data have confirmed the health-promoting effects of fermented foods across a wide range of physiological systems. Notably, Wastyk et al. [6] conducted a landmark 10-week randomized controlled trial in healthy adults and showed that daily intake of fermented foods significantly increased microbiota diversity and reduced the levels of 19 inflammatory markers, including interleukin (IL)-6 and IL-12b, highlighting their immunomodulatory and anti-inflammatory properties.

Other randomized clinical trials have demonstrated diverse health benefits in different populations. Han et al. [79] reported that fermented kimchi improved the levels of metabolic markers and shifted the gut microbiota in overweight women, whereas a kefir-based intervention led to a reduction in inflammatory symptoms in patients with inflammatory bowel disease [80]. In a study of NAFLD and metabolic syndrome, Chen et al. [81] found that yogurt intake significantly improved insulin sensitivity and reduced liver fat accumulation in obese women.

Emerging evidence suggests that fermented foods support cognitive function. In Korea and Japan, fermented milk enriched with *Lactobacillus helveticus* (IDCC3801 or CM4) improved memory and cognitive test scores in elderly and middle-aged participants, respectively, as determined using neuropsychological and biomarker analyses [82]. In patients with Alzheimer’s disease, fermented dairy products containing *Bifidobacterium bifidum*, *Lactobacillus casei*, and *L. acidophilus* have shown potential to mitigate cognitive decline [82].

In addition to brain health, several studies have validated the metabolic benefits of fermented foods. For example, a randomized controlled trial conducted in a high-cardiovascular-risk Mediterranean population revealed that dairy product consumption was associated with a decreased risk of developing type 2 diabetes mellitus over time [83]. Another study by Ecklu-Mensah et al. [63] demonstrated that kombucha consumption in healthy adults modulated the gut microbiota composition and improved inflammatory and metabolic markers, including a reduction in blood pressure and fasting blood glucose level.

Preclinical studies further support the physiological benefits of kombucha, particularly through gut–liver and metabolic axis modulation. In a mouse model of diet-induced obesity and NAFLD, kombucha supplementation significantly improved glucose tolerance, improved hyperinsulinemia, and alleviated hepatic steatosis [84]. These effects were linked to the downregulated expression of pro-inflammatory genes such as *TNF-α* and *SREBP-1*, decreased collagen deposition in liver tissue, and restoration of insulin signaling via AKT phosphorylation, suggesting strong anti-inflammatory and hepatoprotective effects [84].

The above findings were reinforced in a methionine/choline-deficient mouse model of nonalcoholic steatohepatitis (NASH), in which kombucha administration led to a significant reduction in hepatic triglyceride level, inflammation, and fibrosis [85]. Mechanistically, kombucha promoted hepatocyte survival by reducing apoptosis and enhancing cell proliferation, suppressed lipid accumulation through downregulated expression of genes such as *Cd36*, *Pparγ*, *Fas*, and *Srebp1c*, and stimulated β-oxidation via upregulated expression of *Ppargc1α*, *Cpt1*, and *Acox1*. Additionally, kombucha attenuated Hedgehog signaling, a key driver of fibrosis in NASH, by suppressing *Shh*, *Smo*, and *Gli2* expression. Taken together, these hepatoprotective effects highlight the ability of kombucha to modulate both the metabolic and inflammatory pathways. Furthermore, the interaction of kombucha with bile acid receptors such as TGR5 and FXR extends its benefits to lipid regulation and immune modulation.

The consumption of amazake, a traditional Japanese fermented rice beverage, has been shown to reduce serum TNF-α levels and improve symptoms such as muscle cramps and depression in patients with NAFLD and periodontal disease, highlighting the potential of amazake as an anti-inflammatory and quality-of-life-enhancing dietary intervention [86].

The diversity of fermented food types (e.g., kimchi, sauerkraut, miso, sourdough, tempeh, and kombucha) and the broad spectrum of bioactive compounds contribute to their versatile therapeutic applications. According to recent reviews [3,87,88], bioactive metabolites, such as gamma-aminobutyric acid (GABA), EPS, and peptides, produced during fermentation exert direct effects on immune modulation, oxidative stress, and inflammatory pathways. The clinical and preclinical study findings confirm that fermented foods are not only safe but also offer measurable health benefits across the metabolic, cognitive, and immune domains. They highlight the role of fermented foods in preventive and therapeutic nutritional strategies, particularly when guided by microbiome-informed precision tools.

## 7. Conclusions

Fermented foods represent nutritional systems whose health effects extend beyond providing beneficial live microorganisms. Through intricate biochemical transformations, fermentation enhances dietary fiber accessibility and nutrient bioavailability and generates various bioactive metabolites, including organic acids (e.g., acetic and lactic acids), bacteriocins, EPS, vitamins, and bioactive peptides, indirectly supporting gut microbiota-derived SCFA production. The ecological dynamics of fermented food microbiomes influence gut microbiota composition, resilience, and metabolic health via transient microbial and metabolite interactions.

Clinical and preclinical studies substantiate fermented foods’ roles in immune regulation, metabolic health, inflammation control, and cognitive function. However, interindividual variability underscores the importance of personalized nutritional approaches informed by microbiome profiling and precision fermentation technologies.

Future research should focus on standardized clinical trials, mechanistic insights into microbial and metabolite interactions, and innovative fermentation techniques targeting specific health outcomes. Strengthening regulatory frameworks and consumer education will further optimize fermented foods as precise dietary interventions for improved gut and systemic health.

## Figures and Tables

**Table 1:** Summary of fermented foods, dominant microbial taxa, major fermentation metabolites, and associated health-related effects.

Fermented Food	Major Microbes	Key Metabolites	Health-Relevant Effects	Representative References
Yogurt	*Lactobacillus*, *Bifidobacterium*	Lactic acid, EPS, peptides	Improves lactose digestion, enhances gut barrier integrity	[31,32]
Kimchi	*L. plantarum*, *Leuconostoc*	SCFAs, bioactive peptides, vitamins	Anti-inflammatory, immune modulation, improved fiber digestion	[33,34]
Kefir	*Lactococcus*, *Saccharomyces*, *Acetobacter*	SCFAs, EPS, ethanol, peptides	Boosts immunity, modulates microbiota, supports digestion	[35,36]
Sauerkraut	*Leuconostoc*, *Lactobacillus*	SCFAs, bacteriocins, enzymes	Inhibits pathogens, supports gut motility	[37]
Miso	*Aspergillus oryzae*, *Tetragenococcus*	Isoflavones, peptides, enzymes	Antioxidant properties, supports cardiovascular health	[38]
Tempeh	*Rhizopus oligosporus*	Isoflavones, antioxidants, peptides	Anti-inflammatory, antioxidant, gut microbiota support	[39]
Natto	*Bacillus subtilis*	Polyglutamic acid, nattokinase, vitamin K2	Cardiovascular benefits, gut flora modulation	[40]
Fermented Pickles	*Lactobacillus*, *Pediococcus*, *Enterococcus*	Lactic acid, organic acids	Enhances digestion, antimicrobial activity	[41]
Fermented Soy Milk	*Lactobacillus*, *Bifidobacterium*	Isoflavones, SCFAs, peptides	Improves lipid metabolism, balances gut microbiota	[42]
Kombucha	*Brettanomyces*, *Zygosaccharomyces*, *Lachancea*, *Starmerella*, *Saccharomyces*, *Komagataeibacter* (formerly *Gluconacetobacter*), *Acetobacter*, *Gluconobacter*, *Lactobacillus*	Acids, ethanol, glucuronic acid, polyphenols	Detoxification, antioxidant, antimicrobial, gut microbiota modulation	[43,44]

## Data Availability

No new data were created or analyzed in this study. Data sharing is not applicable to this article.
